# Regression of Albuminuria and Hypertension and Arrest of Severe Renal Injury by a Losartan-Hydrochlorothiazide Association in a Model of Very Advanced Nephropathy

**DOI:** 10.1371/journal.pone.0056215

**Published:** 2013-02-19

**Authors:** Simone Costa Alarcon Arias, Carla Perez Valente, Flavia Gomes Machado, Camilla Fanelli, Clarice Silvia Taemi Origassa, Thales de Brito, Niels Olsen Saraiva Camara, Denise Maria Avancini Costa Malheiros, Roberto Zatz, Clarice Kazue Fujihara

**Affiliations:** 1 Laboratory of Renal Pathophysiology (LIM-16), Renal Division, Department of Clinical Medicine, Faculty of Medicine, University of São Paulo, São Paulo, Brazil; 2 Department of Pathology, Faculty of Medicine, University of São Paulo, São Paulo, Brazil; 3 Laboratory of Immunology, Nephrology Division, Faculty of Medicine, Federal University of São Paulo, São Paulo, Brazil; University of Florida, United States of America

## Abstract

Treatments that effectively prevent chronic kidney disease (CKD) when initiated early often yield disappointing results when started at more advanced phases. We examined the long-term evolution of renal injury in the 5/6 nephrectomy model (Nx) and the effect of an association between an AT-1 receptor blocker, losartan (L), and hydrochlorothiazide (H), shown previously to be effective when started one month after Nx. Adult male Munich-Wistar rats underwent **Nx,** being divided into four groups: **Nx+V**, no treatment; **Nx+L**, receiving L monotherapy; **Nx+LH**, receiving the L+H association (LH), and **Nx+AHHz**, treated with the calcium channel blocker, amlodipine, the vascular relaxant, hydralazine, and H. This latter group served to assess the effect of lowering blood pressure (BP). Rats undergoing sham nephrectomy (**S**) were also studied. In a first protocol, treatments were initiated 60 days after Nx, when CKD is at a relatively early stage. In a second protocol, treatments were started 120 days after Nx, when glomerulosclerosis and interstitial fibrosis are already advanced. In both protocols, L treatment promoted only partial renoprotection, whereas LH brought BP, albuminuria, tubulointerstitial cell proliferation and plasma aldosterone below pretreatment levels, and completely detained progression of renal injury. Despite normalizing BP, the AHHz association failed to prevent renal damage, indicating that the renoprotective effect of LH was not due to a systemic hemodynamic action. These findings are inconsistent with the contention that thiazides are innocuous in advanced CKD. In Nx, LH promotes effective renoprotection even at advanced stages by mechanisms that may involve anti-inflammatory and intrarenal hemodynamic effects, but seem not to require BP normalization.

## Introduction

Although several experimental treatments intended to detain the progression of CKD have been proposed in the past few decades, only a small minority could be translated into clinical practice. One possible reason for this is that, in general, treatment is initiated in concomitance with the onset of the disease or a few days thereafter, artificially increasing the effectiveness of therapy, since in this manner the pathogenic factors involved are more easily neutralized. Far less encouraging results are obtained if treatment is initiated at later stages, when the much more complex interaction between these factors would require more vigorous therapy and the association of two or more drugs [1]–[3].

Five-sixths renal ablation (Nx), a widely employed model of chronic kidney disease (CKD), is characterized by severe glomerular and interstitial injury, accompanied by marked hypertension and renal functional loss. Both hemodynamic and inflammatory phenomena are thought to participate in the pathogenesis of renal injury in the Nx model [4], [5]. Accordingly, treatment with inhibitors of the renin-angiotensin system (RAS) provides significant renoprotection in the Nx model, as well as in other CKD models and in clinical CKD [4], [6]–[12]. However, renoprotection afforded by these compounds is far from complete [1], [13], [14], which has prompted their association with drugs with different mechanisms of action, such as anti-inflammatory or antilymphocytic agents [1], [3]. Nevertheless, chronic associations of RAS inhibitors with potentially toxic drugs are unlikely to be translated into clinical practice.

We showed previously [15] that an association between the Angiotensin-II (AII) receptor blocker, losartan (L), and the thiazide diuretic, hydrochlorotiazide (H), started 30 days after Nx, normalized blood pressure and albuminuria, and provided complete renoprotection for at least 7 months. These results did not support the established concept that thiazide diuretics are ineffective when renal function has declined to 1/3 of normal or less [16], as in the Nx model [4], [5], [17]. However, these findings may not be applicable to the clinical setting. First, although the Nx model is presumed to mimic advanced CKD because the nephron number is so drastically reduced, it may not reflect the real clinical situation, because in patients with advanced CKD severe nephron loss is due to a long process of inflammation and fibrosis, while in the Nx model nephron reduction is an immediate consequence of surgical removal and, even after 30 days, renal inflammation and fibrosis are still relatively limited. Second, the striking renoprotection afforded by the L+H association might merely reflect normalization of blood pressure [15], and in this case could be entirely attributable to amelioration of the hemodynamic strain to the renal microcirculation.

In the present study, we investigated whether the L+H association would still arrest the progression of renal injury at more advanced stages of CKD in the Nx model, when extensive renal fibrosis is already present, thereby mimicking more closely the situation prevailing in advanced human CKD. In addition, we sought to determine whether control of systemic hypertension would play a central role in a possible renoprotective effect of the combined L+H treatment in this setting.

## Methods

Two hundred thirty-two adult male Munich-Wistar rats, weighing between 220 and 260 g were utilized in this study. All rats were obtained from a local facility at the Faculty of Medicine, University of São Paulo. All experimental procedures were specifically approved by the local Research Ethics Committee (Comissão de Ética para Análise de Projetos de Pesquisa do Hospital das Clínicas da Faculdade de Medicina da Universidade de São Paulo, CAPPesq, under process n° 0689/08), and developed in strict conformity with our institutional guidelines and with international standards for manipulation and care of laboratory animals. All rats were monitored daily for body weight and general condition. Rats that were in bad condition, presumably due to end-stage renal failure were euthanized by an overdose of anesthetic. Five-sixths renal ablation (Nx) was performed in a single-step procedure after ventral laparotomy under anesthesia with ketamine 50 mg/kg and xylazine 10 mg/kg im. The right kidney was removed and two or three branches of the left renal artery were ligated, resulting in the infarction of two-thirds of the left kidney. Sham-operated rats underwent anesthesia and manipulation of the renal pedicles, without any removal of renal mass. After surgery, all animals received enrofloxacin and, after full recovery, were given free access to tap water, fed regular rodent chow containing 0.5 Na and 22% protein (Nuvital Labs, Curitiba, Brazil), and kept at 23±1°C and 60±5% relative air humidity, under an artificial 12–12 hour light/dark cycle.

The possibility of regression or prevention of renal injury was analyzed in 2 protocols representing a moderately advanced (Protocol 1) and a very advanced (Protocol 2) stage of chronic nephropathy.

### Protocol 1

Sixty days after Nx, tail-cuff pressure (TCP) and daily urinary albumin excretion (U_alb_V) were determined in all Nx rats. Those failing to increase TCP above 170 mmHg or U_alb_V above 40 mg/day were not included in the study. Fifteen Nx rats were killed at this time and were used as pretreatment control subjects (group Nx_pre_). The remaining 74 Nx rats were divided into 4 experimental groups: Nx+V (n = 22); Nx+L (n = 15), Nx receiving losartan potassium (L), 50 mg/kg diluted in the drinking water; Nx+LH (n = 17), receiving L 50 mg/kg plus hydrochlorothiazide (H) 6 mg/kg diluted in the drinking water; Nx+AHHz (n = 20), receiving amlodipine besylate 5 mg/kg plus H 6 mg/kg plus hydralazine chloride (Hz) 12 mg/kg. This last group was included to evaluate the degree of renal protection that would be afforded by decreasing TCP to a similar extent as in group LH, without suppressing the renin-angiotensin system. Nx rats were distributed in such a way that initial body weight (BW), TCP and U_alb_V were similar among experimental groups. All treatments were maintained for 90 days. A group of 15 Sham-operated rats receiving no treatment was followed for the same time.

### Protocol 2

One hundred twenty days after Nx, TCP and U_alb_V were determined in all Nx rats. Twenty Nx rats were utilized at this time as pretreatment controls (Nx_pre_). The remaining 85 Nx rats were distributed in the same way as for Protocol 1, among groups: Nx+V (n = 26); Nx+L (n = 19); Nx+LH (n = 20); and Nx+AHHz (n = 20), treatments being maintained for 90 days. A group of 23 untreated sham-operated rats was followed for the same time.

### Long-term Studies and Preparation of Renal Tissue for Histological Evaluation

Monthly U_alb_V was evaluated by radial immunodiffusion and TCP was measured using an optoelectronic automated device (Visitech Systems, Apex, NC) under light restraining and after light warming. To avoid any interference of stress, all rats were preconditioned to the procedure, and were invariably calm at the time of TCP determination. In addition, TCP was taken as the average of at least three consecutive measurements that varied by no more than 2 mmHg, to ensure that BP was stable before readings. At the end of the study, blood glucose (BG) and plasma triglycerides (Tg) were measured in blood taken from a tail vein after 12-hour fasting. On the following day, rats were anesthetized with ketamine, 50 mg/kg and xylazine 10 mg/kg im, and blood was collected from the abdominal aorta for measurement of serum creatinine (S_Cr_), aldosterone (ALDO) and potassium (K^+^) concentration. The kidneys were then retrogradely perfusion-fixed through the abdominal aorta with Dubosq-Brazil solution after a brief washout with saline to remove blood from the renal vessels. After weighing, two midcoronal renal slices were postfixed in buffered 4% formaldehyde and embedded in paraffin using conventional sequential techniques. Histomorphometric and immunohistochemical analyses of the renal tissue were performed in 4-µm-thick sections.

### Biochemical Analyses

Tg and BG were measured in blood samples obtained from a tail vein Tg was measured using a commercially available enzymatic kit (Labtest Diagnostica, São Paulo, Brazil). BG concentration was determined using a reﬂectometric method (Advantage, Roche Diagnostics, USA).

S_Cr_ was determined using a commercially available kit (Labtest Diagnostica, São Paulo, Brazil). A radioimmunoassay kit (Diagnostic Systems Laboratories, Inc., Texas, USA) was used to determine ALDO. Serum K^+^ was measured using an electrolyte analyzer (AVL Medical Instruments).

### Histomorphometric Analysis

Morphometric evaluations were always performed in a blinded manner by a single observer. The extent of glomerular injury was estimated by determining the frequency of glomeruli with sclerotic lesions, as described previously [9], [18] in sections stained by the periodic acid-Schiff reaction. At least 120 glomeruli were examined for each rat. The percentage of the renal cortical area occupied by interstitial tissue, used as a measure of the degree of interstitial expansion (%INT), was estimated in 25 consecutive microscopic fields of Masson-stained sections by a point-counting technique [19], at a final magnification of 100×, under a 144-point grid.

### Immunohistochemical Analysis

Immunohistochemistry was performed on 4-µm-thick sections, mounted on glass slides precoated with 2% silane. Sections were deparaffinized and rehydrated by conventional techniques, then heated in citrate buffer for antigen retrieval and incubated overnight with the primary antibody at 4°C. For the negative control experiments, incubation with the primary antibody was not performed. The following primary antibodies were used: monoclonal mouse anti-rat ED-1 antibody for macrophage detection (Serotec, Oxford, United Kingdom); monoclonal mouse anti-rat proliferating cell nuclear antigen (PCNA) (Dako, Glostrup, Denmark); monoclonal mouse anti-alpha-smooth muscle actin (α-SMA) (Sigma, Missouri, USA); polyclonal rabbit anti-human AII (Peninsula Laboratories, San Carlos, USA), polyclonal rabbit Anti-Collagen I (Abcam, Cambridge, United Kingdom) and polyclonal rabbit anti-rat thiazide-sensitive Na-Cl cotransporter, NCC (Chemicon International, Temecula, USA).

For ED-1 detection, sections were preincubated with 5% normal rabbit serum to prevent nonspecific binding, then incubated overnight at 4°C with the primary antibody diluted in bovine serum albumin (BSA) at 1%. After rinsing with Tris-buffered saline (TBS), sections were incubated with an appropriate secondary antibody, then with an alkaline phosphatase anti-alkaline phosphatase (APAAP) complex (Dako, Glostrup, Denmark). Sections were developed with a fast-red dye solution (Sigma-Aldrich, Saint Louis, MO). For AII and α-SMA detection a streptavidin-biotin complex for alkaline phosphatase (DakoCytomation, Glostrup, Denmark) was used. Nonspecific binding was prevented with normal horse serum diluted at 1∶20 in nonfat milk at 2% in TBS. Primary antibodies for AII and α-SMA were diluted in BSA at 1∶400 and 1∶800, respectively. Sections were developed in the same manner as for ED-1 detection. For assessment of PCNA-positive cells and the percentage of the renal area occupied by collagen I, sections were pretreated with 30% hydrogen peroxide in methanol and preincubated with normal horse serum as described. The primary antibodies were diluted at 1∶100 (PCNA) and 1∶200 (collagen I), in nonfat milk at 2% in TBS. The EnVision Labelled Polymer for peroxydase (Dako, Glostrup, Denmark) was used before development with DAB substrate (Dako, Glostrup, Denmark).

Double immunostaining was used to visualize the proliferation activity of distal convoluted tubules (DCT). Identification of DCT was performed by detection of NCC. Sections were pretreated with 30% hydrogen peroxide in methanol and preincubated with avidin and biotin blocking solutions (Vector, Burlingame, CA). Nonspecific staining was then prevented with normal goat serum diluted at 5% in BSA at 1% in TBS. Sections were incubated overnight with primary antibody against NCC diluted at 1% in BSA at 1% in TBS. Appropriate biotinylated secondary antibody was applied and Streptavidin-AP solution (DakoCytomation, Glostrup, Denmark) was used, followed by development with fast-red dye solution (Sigma-Aldrich, Saint Louis, MO). Sections were then preincubated once again with avidin and biotin blocking solutions, followed by prevention of nonspecific staining with a mixture of normal horse and rabbit sera diluted at 2 and 5%, respectively, in 2% nonfat milk in TBS. Sections were then incubated overnight with the primary antibody against PCNA, 0.01% in a solution containing 1% BSA and 2% nonfat milk diluted in TBS. Appropriate biotinylated secondary antibodies were applied and the LSAB-HRP kit (DakoCytomation, Glostrup, Denmark) was used for PCNA detection. Sections were developed with DAB substrate (Dako, Glostrup, Denmark). All sections were counterstained with Mayer’s hematoxylin, dehydrated and covered with Permount Mounting Media (Thermo Fisher Scientific, New Jersey, USA).

The renal density of macrophages, proliferating cells and AII positive cells was evaluated in a blinded manner at 200× magnification. For each section, 50 microscopic fields (corresponding to a total area of 1.6 mm^2^) were examined. The percentage of cortical interstitial area occupied by α-SMA was estimated by the same point-counting technique employed to evaluate %INT, excluding positively stained blood vessels, while interstitial area occupied by collagen I was measured with an image processing software (Image Pro Plus®, version 7.01).

### Statistical Analysis

Differences among different groups were analyzed using one-way analysis of variance (ANOVA) with pairwise post-test comparisons by the Neuman-Keuls method [20]. Since U_alb_V, ALDO, AII and PCNA+NCC rates exhibited a strong non-Gaussian distribution, log transformation of these data was performed prior to statistical analysis. Mortalities were analyzed using a Kaplan-Meier approach. *p* values less than 0.05 were considered significant. Results are presented as Mean±1 SE. Calculations were performed using Prism® 4.0 (GraphPad® Software, USA).

## Results

**Figure 1:**
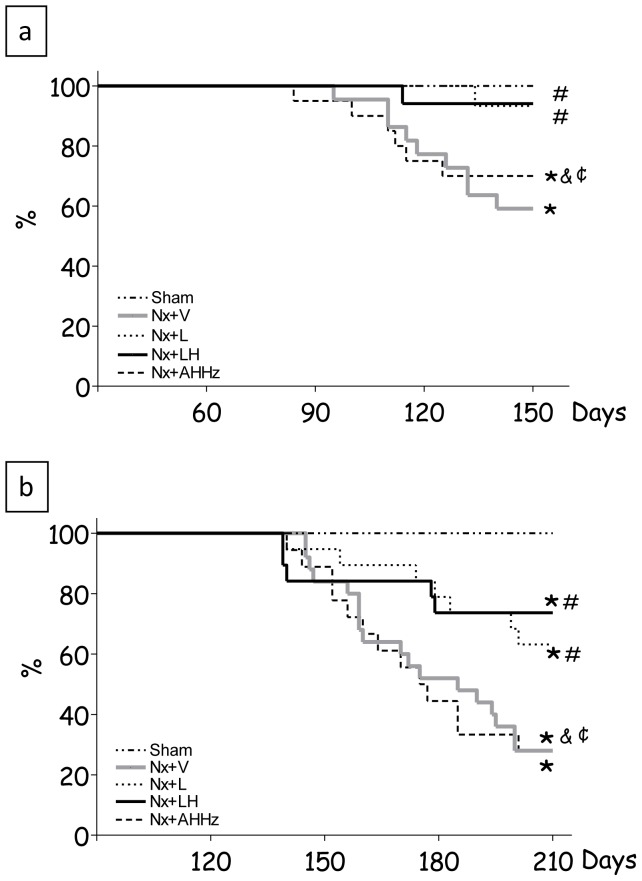
Percent Survival in Protocol 1 (a) and Protocol 2 (b). S, Sham-operated; Nx+V, untreated Nx; Nx+L, losartan-treated Nx; Nx+LH, Nx treated with losartan and hydrochlorothiazide; Nx+AHHz, Nx treated with amlodipine, hydrochlorothiazide, and hydralazine. *, p<0.05 vs. Sham; **^#^**, p<0.05 vs. Nx+V; **^&^** p<0.05 vs. Nx+L and **^¢^**, p<0.05 vs. Nx+LH.

Survival data for Protocol 1 are shown in Figure 1a. In group Nx+V, mortality 150 days after renal ablation was 41%, whereas in the groups treated with L and LH it was reduced to 7% and 6%, respectively (p<0.05 vs. Nx+V). AHHz treatment promoted no improvement in survival, which reached 30% (p>0.05 vs. Nx+V). In Protocol 2 (Fig. 1b), the mortality rate at 210 days after nephrectomy was 70%, while in Groups Nx+L and Nx+LH rates were 40% and 25%, respectively (p<0.05 vs. Nx+V). Treatment with AHHz did not attenuate mortality, which remained at 69% (p>0.05 vs. Nx+V). No deaths occurred in Group S.

Body weights observed in Protocol 1 are shown in Table 1. In all Nx groups, body growth was stunted compared with the S group (p<0.05). There was no significant difference among the treated groups. In Protocol 2 (Table 2), body weight was also reduced compared with S, again without differences among treated groups.

**Table 1:** Functional and morphologic parameters in Protocol 1 (treatments started 60 days after renal ablation).

	BW	S_cr_	K^+^	%INT	M∅	AII+	BG	Tg
**Sham**	*395±7*	*0.6±0.1*	*4.9±0.1*	*0.1±0.1*	*20±2*	*0.5±0.1*	*97±2*	*64±4*
**Nx_pre_**	*284±6* a	*1.0±0.1* a	*5.8±0.1* a	*3.0±0.3* a	*128±17* a	*6.7±1.2* a	*98±3*	*70±3*
**Nx+V**	*297±12* a	*1.7±0.1^ab^*	*5.8±0.3* a	*4.3±0.4* a	*191±15^ab^*	*16.0±1.8^ab^*	*104±3*	*92±7^ab^*
**Nx+L**	*314±9* a	*1.3±0.1^ac^*	*6.0±0.1* a	*3.8±0.6* a	*131±13^ac^*	*13.9±1.3^ab^*	*108±2* a	*75±6*
**Nx+LH**	*296±6* a	*1.1±0.1^ac^*	*5.6±0.1* a	*3.0±0.3* a	*113±11^ac^*	*9.5±1.5^acd^*	*105±2*	*64±4* c
**Nx+AHHz**	*306±10* a	*1.5±0.2^abe^*	*4.7±0.1^bcde^*	*4.1±0.4* a	*180±20^abde^*	*14.1±1.5^abe^*	*108±3* a	*79±8*

BW, body weight, g; S_cr_, serum creatinine, mg/dL; K^+^, serum potassium, mmol/L; %INT, fractional cortical interstitial area; MØ, density of tubulointerstitial macrophages, cells/mm^2^; AII+, density of tubulointerstitial cells staining positively for AII, cells/mm^2^; BG, blood glucose, mg/dL; Tg, triglycerides, mg/dL; S, Sham-operated; Nx_pre_, pretreatment Nx (60 days after renal ablation); Nx+V, untreated Nx; Nx+L, losartan-treated Nx; Nx+LH, Nx treated with losartan+hydrochlorothiazide; Nx+AHHz, Nx treated with amlodipine, hydrochlorothiazide, and hydralazine. Group Nx+V and all Nx treated groups studied 150 days after renal ablation. Results expressed as Mean ±1 SE;

a p<0.05 vs. Sham;

b p<0.05 vs. Nx_pre_;

c p<0.05 vs. Nx+V;

d p<0.05 vs. Nx+L and

e p<0.05 vs. Nx+LH.

**Table 2:** Functional and morphologic parameters in Protocol 2 (treatments started 120 days after renal ablation).

	BW	S_cr_	K^+^	%INT	M∅	AII+	BG	Tg
**Sham**	*406±7*	*0.6±0.1*	*4.9±0.1*	*0.1±0.1*	*22±2*	*0.7±0.1*	*97±1*	*54±4*
**Nx_pre_**	*305±6* a	*1.4±0.1* a	*5.5±0.1* a	*4.2±0.5* a	*188±18* a	*12.7±1.8* a	*94±2*	*77±2* a
**Nx+V**	*279±6* a	*2.5±0.1^ab^*	*5.9±0.2* a	*7.2±0.5^ab^*	*250±18^ab^*	*19.8±2.0^ab^*	*123±10^ab^*	*95±5* a
**Nx+L**	*293±9* a	*2.1±0.2^ab^*	*6.2±0.2^ab^*	*6.9±0.7^ab^*	*189±12^ac^*	*17.3±1.4^ab^*	*114±5^ab^*	*84±10* a
**Nx+LH**	*283±7* a	*1.6±0.1^acd^*	*5.7±0.1^ad^*	*4.0±0.5^acd^*	*149±13^ac^*	*11.9±1.7^acd^*	*116±2^ab^*	*64±5* c
**Nx+AHHz**	*291±8* a	*2.1±0.2^abe^*	*5.1±0.2^bcde^*	*6.5±0.6^abe^*	*188±14^ac^*	*20.6±1.4^abe^*	*132±6^ab^*	*100±15^ae^*

BW, body weight, g; S_cr_, serum creatinine, mg/dL; K^+^, serum potassium, mmol/L; %INT, fractional cortical interstitial area; MØ, density of tubulointerstitial macrophages, cells/mm^2^; AII+, density of tubulointerstitial cells staining positively for AII, cells/mm^2^; BG, blood glucose, mg/dL; Tg, triglycerides, mg/dL; S, Sham-operated; Nx_pre_, pretreatment Nx (120 days after renal ablation); Nx+V, untreated Nx; Nx+L, losartan-treated Nx; Nx+LH, Nx treated with losartan+hydrochlorothiazide; Nx+AHHz, Nx treated with amlodipine, hydrochlorothiazide, and hydralazine. Group Nx+V and all Nx treated groups studied 210 days after renal ablation. Results expressed as Mean ±1 SE;

a p<0.05 vs. Sham;

b p<0.05 vs. Nx_pre_;

c p<0.05 vs. Nx+V;

d p<0.05 vs. Nx+L and

e p<0.05 vs. Nx+LH.

**Figure 2:**
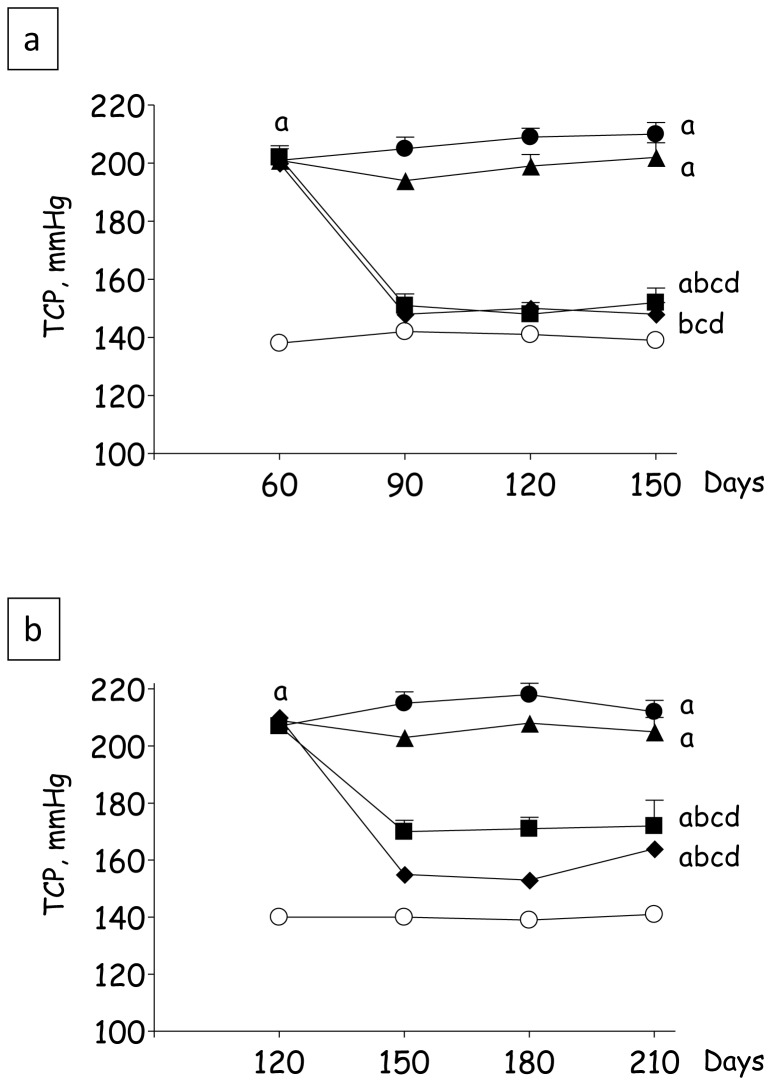
Tail-cuff pressures (TCP, mmHg) in Protocol 1 (a) and Protocol 2 (b). S, Sham-operated (clear circles); Nx_pre_, pretreatment Nx (60 or 120 days after renal ablation); Nx+V (filled circles), untreated Nx; Nx+L (triangles), losartan-treated Nx; Nx+LH (diamonds), Nx treated with losartan+hydrochlorothiazide; Nx+AHHz (squares), Nx treated with amlodipine, hydrochlorothiazide, and hydralazine. Results expressed as Mean ± SE. ^a^, p<0.05 vs. Sham; ^b^, p<0.05 vs. Nx_pre_; ^c^, p<0.05 vs. Nx+V; ^d^, p<0.05 vs. Nx+L and ^e^, p<0.05 vs. Nx+LH.

Figure 2a shows the behavior of TCP over time in Protocol 1. Group Nx+V exhibited hypertension along the entire period of observation, reaching 210±4 mmHg at the end of the study (p<0.05 vs. S). Treatment with L had little effect on TCP, which was comparable to that observed in Group Nx+V 150 days after nephrectomy (p>0.05). By contrast, TCP was brought to normal along the whole study in rats receiving the LH treatment, which remained normotensive even at 150 days after renal ablation. TCP in animals treated with AHHz was indistinguishable from that in Group Nx+LH at the end of the study. Results for Protocol 2 are shown in Figure 2b. Nx+V animals remained severely hypertensive along the entire study, TCP reaching 212±4 mmHg 210 days after ablation (p<0.05 vs S). L treatment failed to lower TCP, which was similar to that observed in untreated rats at the end of the observation period. Again, Groups Nx+LH and Nx+AHHz exhibited low values for TCP along the whole study (p<0.05 vs. Nx+V and Nx+L).

**Figure 3:**
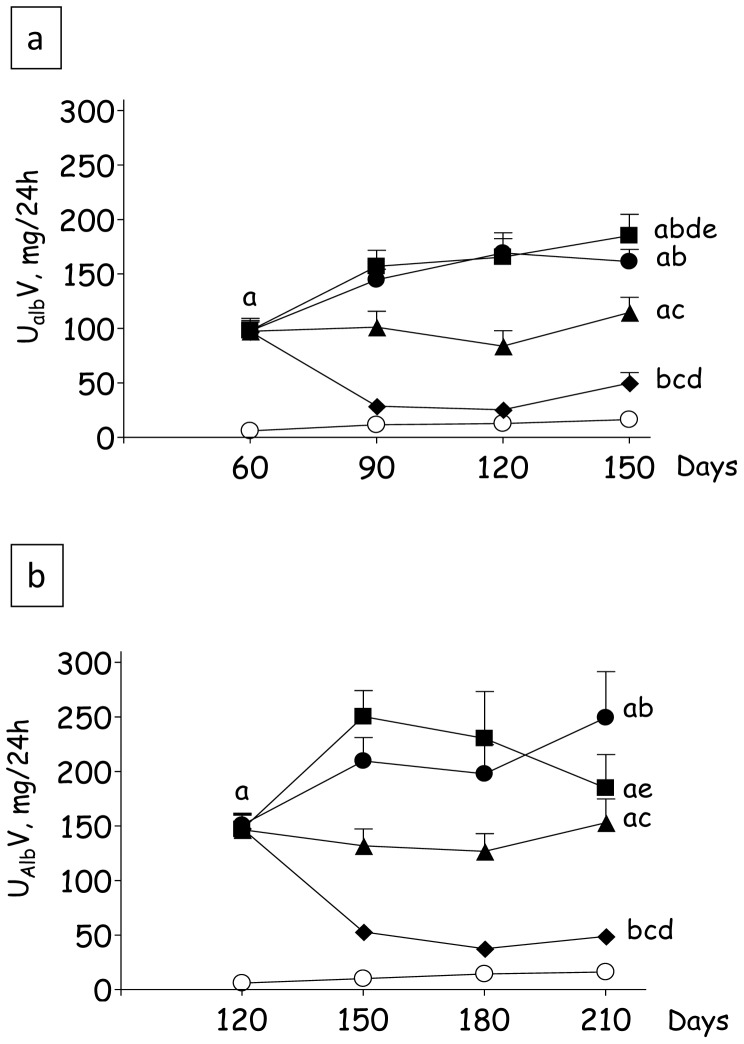
Urinary albumin excretion rates (U_alb_V, mg/24 h) in Protocol 1 (a) and Protocol 2 (b). S, Sham-operated (clear circles); Nx_pre_, pretreatment Nx (60 or 120 days after renal ablation); Nx+V (filled circles), untreated Nx; Nx+L (triangles), losartan-treated Nx; Nx+LH (diamonds), Nx treated with losartan+hydrochlorothiazide; Nx+AHHz (squares), Nx treated with amlodipine, hydrochlorothiazide, and hydralazine. Results expressed as Mean ± SE. ^a^, p<0.05 vs. Sham; ^b^, p<0.05 vs. Nx_pre_; ^c^, p<0.05 vs. Nx+V; ^d^, p<0.05 vs. Nx+L and ^e^, p<0.05 vs. Nx+LH.

The variation of U_alb_V with time in Protocol 1 is shown in Figure 3a. Nx+V animals showed a progressive increase in U_alb_V, reaching values tenfold higher than in S at 150 days (p<0.05 vs. S). In Group Nx+L, U_alb_V remained markedly elevated along the study, although final values were nearly 30% lower than in Group Nx+V (p<0.05). By contrast, combined LH treatment promoted U_alb_V regression, keeping it close to S values until the end of the study (p>0.05), although it should be noted that, since the nephron number had been reduced to 1/6th of normal, albuminuria per nephron was still higher in this group compared to S. Treatment with AHHz was unable to lower U_alb_V, which was always similar to that seen in Group Nx+V. The evolution of U_alb_V in Protocol 2 is represented in Figure 3b. Again, U_alb_V was always elevated in Group Nx+V, being lowered by a small but significant amount by L treatment. Triple AHHz therapy had little effect on U_alb_V. Even at this advanced stage, the LH treatment reduced U_alb_V to levels that approached those observed in S, remaining at these low levels until the end of the observation period (p>0.05 vs. S).

Serum creatinine concentrations (S_Cr_) for Protocol 1 are shown in Table 1. Sixty days after renal ablation (Group Nx_pre_) S_Cr_ was twice as high as in Group S (p<0.05). At the end of Protocol 1, 150 days after renal ablation, S_cr_ was increased further (p<0.05 vs. S and Nx_pre_), indicating progression of the nephropathy. Treatment with L attenuated the loss of renal function, maintaining S_cr_ at levels comparable to those observed in the pretreatment group (p>0.05 vs Nx_pre_ and p<0.05 vs. Nx+V). Similar functional protection was observed with the LH association (p>0.05 vs Nx+L). The AHHz scheme had no effect on S_Cr_, which increased over time and was indistinguishable from the Nx+V value at the end of the study. In Protocol 2 (Table 2) S_cr_ was increased in the Nx_pre_ group, which was studied 120 days after nephrectomy (p<0.05 vs S). Animals in Group Nx+V showed severe loss of renal function 210 days after renal ablation, as indicated by a marked increase in S_Cr_ (p<0.05 vs. Nx_pre_). Treatment with L or AHHz had little effect on S_Cr_, which attained levels similar to those seen in Group Nx+V at the end of the study (p>0.05). By contrast, rats treated with the LH combination exhibited final S_Cr_ levels that were significantly lower than in the remaining groups, and similar to those verified in Group Nx_pre_ (p>0.05).

Serum K^+^ concentrations for Protocol 1 are shown in Table 1. Sixty days after renal ablation (Group Nx_pre_) serum K^+^ was significantly increased compared to S. Animals in group Nx, at both 60 and 150 days after nephrectomy, showed an increase in serum potassium (p<0.05 vs S). Treatments with L or LH had no effect on serum K^+^ at the end of the period of observation. The AHHz regimen strongly decreased serum K^+^, which was similar to that observed in the S group 150 days after renal ablation. Values for Protocol 2 are shown in Table 2. Again, hyperkalemia was seen in the Nx_pre_ group, 120 days after nephrectomy (p<0.05 vs. S), and in Group Nx+V, examined at the end of the study. Treatment with L aggravated hyperkalemia, serum K^+^ reaching values significantly higher than those observed in the Nx_pre_ group (p<0.05). In animals receiving LH therapy, serum K^+^ was ameliorated compared to the Nx+L group (p<0.05), being similar to that seen in the Nx_pre_ group (p>0.05). As observed in Protocol 1, treatment with AHHz significantly reduced serum K^+^, which was brought to levels similar to those observed in the S group (p>0.05).

**Figure 4:**
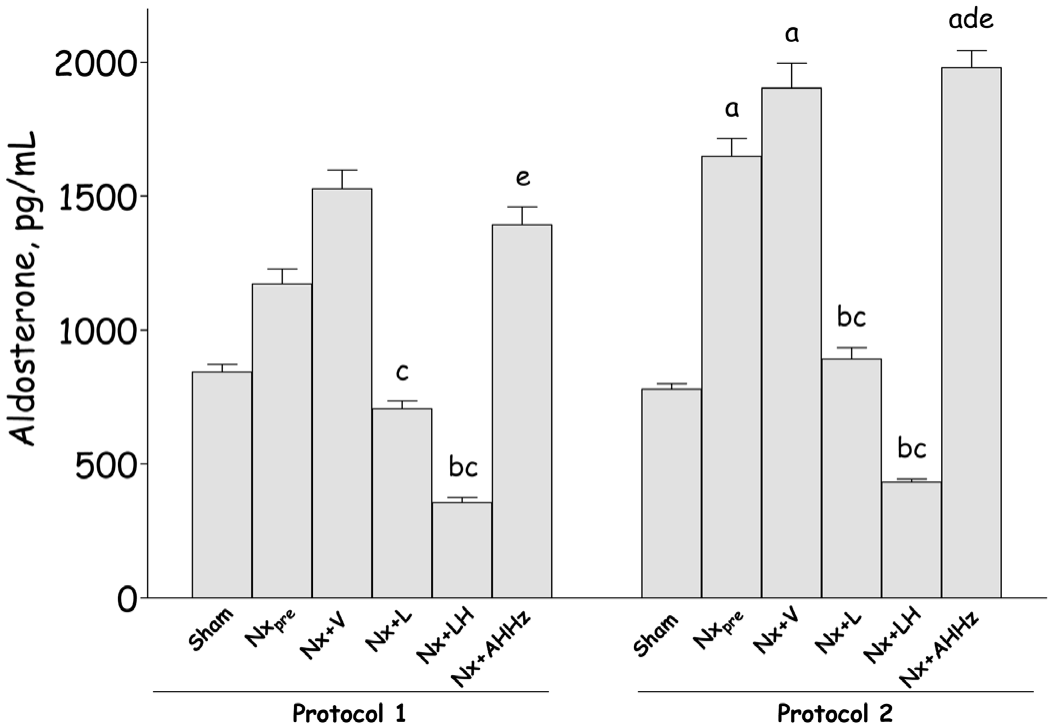
Plasma aldosterone (ALDO, pg/mL) in Protocol 1 and Protocol 2. S, Sham-operated; Nx_pre_, pretreatment Nx (60 or 120 days after renal ablation); Nx+V, untreated Nx; Nx+L, losartan-treated Nx; Nx+LH, Nx treated with losartan+hydrochlorothiazide; Nx+AHHz, Nx treated with amlodipine, hydrochlorothiazide, and hydralazine. Results expressed as Mean ± SE. ^a^, p<0.05 vs. Sham; ^b^, p<0.05 vs. Nx_pre_; ^c^, p<0.05 vs. Nx+V; ^d^, p<0.05 vs. Nx+L and ^e^, p<0.05 vs. Nx+LH.

The values for aldosterone (ALDO) in both protocols are shown in Figure 4. In Protocol 1, ALDO was numerically higher in Nx animals at 150 days, compared to 60 days, after renal ablation. Treatment with L significantly reduced the final concentration of ALDO (p<0.05 vs. Nx+V). The LH association reduced ALDO to values lower than in Nx_pre_ (p<0.05 vs. Nx_pre_, Nx+V and p>0.05 vs. S). Triple therapy promoted no change in ALDO concentration, which remained comparable to that in Group Nx+V (p>0.05). In Protocol 2, the pretreatment ALDO concentration (Group Nx_pre_) was significantly higher than in Group S (p<0.05), a difference that persisted in Group Nx+V. Monotherapy with L promoted a significant reduction compared to Group Nx+V (p<0.05). LH treatment drastically reduced circulating ALDO below pretreatment levels (p<0.05 vs. Nx_pre_, Nx+V and p>0.05 vs. S). In Group Nx+AHHz serum ALDO was similar to that in Group Nx+V.

**Figure 5:**
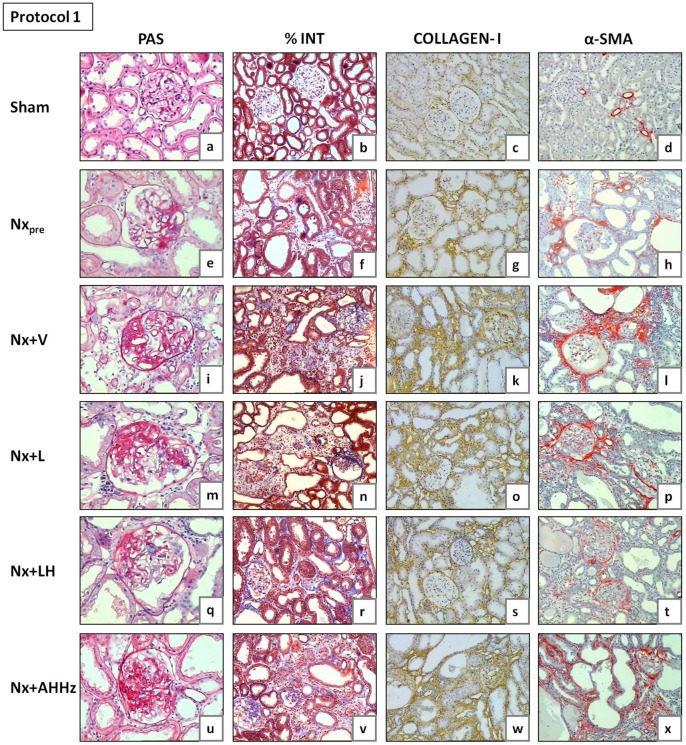
Representative microphotographs of renal tissue from Nx_pre_ (60 days after renal ablation) and from all other groups (150 days after renal ablation) in Protocol 1. PAS, Periodic Acid-Schiff; %INT, percent cortical area occupied by interstitium in sections stained with Masson trichrome; α-SMA, alpha-smooth muscle actin.

**Figure 6:**
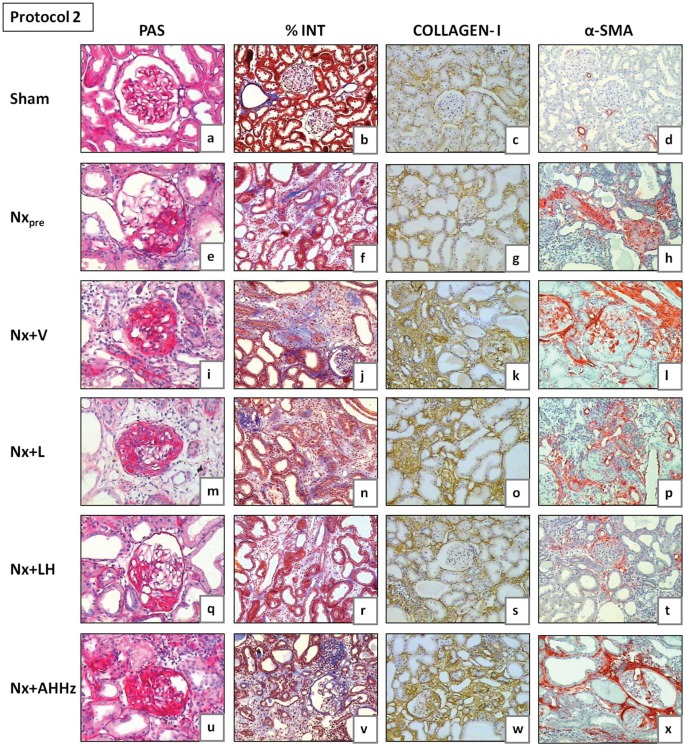
Representative microphotographs of renal tissue from Nx_pre_ (120 days after renal ablation) and from all other groups (210 days after renal ablation) in Protocol 1. PAS, Periodic Acid-Schiff; %INT, percent cortical area occupied by interstitium in sections stained with Masson trichrome; α-SMA, alpha-smooth muscle actin.

**Figure 7:**
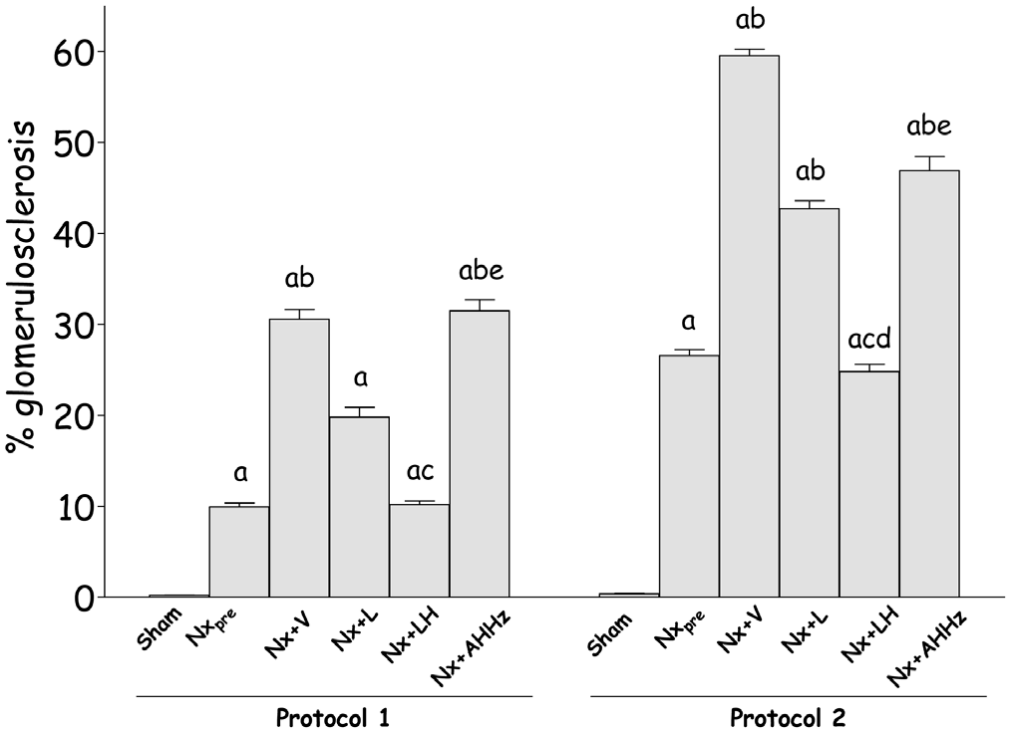
Percent glomerulosclerosis (%GS) in Protocol 1 and Protocol 2. S, Sham-operated; Nx_pre_, pretreatment Nx (60 days or 120 after renal ablation); Nx+V, untreated Nx; Nx+L, losartan-treated Nx; Nx+LH, Nx treated with losartan+hydrochlorothiazide; Nx+AHHz, Nx treated with amlodipine, hydrochlorothiazide, and hydralazine. Results expressed as Mean ± SE. ^a^, p<0.05 vs. Sham; ^b^, p<0.05 vs. Nx_pre_; ^c^, p<0.05 vs. Nx+V; ^d^, p<0.05 vs. Nx+L and ^e^, p<0.05 vs. Nx+LH.

Representative glomeruli seen at the end of Protocol 1 (150 days after renal ablation) are shown in Fig. 5 (a, e, i, m, q and u), while analogous microphotographs for Protocol 2 are shown in Fig. 6 (a, e, i, m, q and u). The frequency of sclerotic glomeruli (% GS) in both protocols is given in Figure 7. In Protocol 1, almost 10% of glomeruli exhibited sclerotic lesions 60 days after renal ablation (Group Nx_pre_). Glomerular injury progressed in untreated animals, %GS exceeding 30% in Group Nx+V 150 days after nephrectomy (p<0.05 vs. Nx_pre_). Treatment with L alone partially prevented the progression of glomerular lesions, although %GS in Group Nx+L was not significantly different from that seen in untreated rats (p>0.05 vs. Nx_pre_ and Nx+V). Glomerular protection was evident in the group receiving the LH association, in which the value for %GS was nearly identical to that observed in Group Nx_pre_. In the group treated with the AHHz association, the frequency of glomerular sclerotic lesions was not limited, progressing in a similar fashion as in the untreated group. In Protocol 2, %GS already reached 27% 120 days after renal ablation (Group Nx_pre_). After 210 days post-ablation, almost 60% of glomeruli exhibited sclerotic lesions (p<0.05 vs. Nx_pre_). Treatment with L failed to prevent the progression of %GS, which reached values that were lower than those found in untreated rats, but significantly higher than pretreatment values. In rats treated with LH, %GS reached final values that were significantly lower than in Group Nx+V or Nx+L, and similar to those seen in Group Nx_pre_. In animals treated with AHHz, %GS behaved in a similar manner as in the untreated group.

Representative microphotographs of cortical interstitial area are shown in Fig. 5 (b, f, j, n, r, and v) for Protocol 1 and Fig. 6 (b, f, j, n, r, and v) for Protocol 2. Quantitative analysis of the percent cortical interstitium for Protocol 1 (%INT) is shown in Table 1. Group Nx_pre_ exhibited high %INT values compared to S (p<0.05). Little progression was seen in untreated rats 150 days after ablation (p<0.05 vs. Nx_pre_). None of the treatments had a significant effect on %INT at this phase. Table 2 shows the quantitative analysis of %INT for Protocol 2. Values for %INT were significantly higher in Group Nx_pre_ in comparison with S. At 210 days post-ablation, %INT was significantly increased in Group Nx+V compared with pretreatment values (p<0.05 vs. Nx_pre_). L monotherapy was unable to attenuate the progression of interstitial expansion, %INT reaching similar values as in Group Nx+V. By contrast, the LH association completely prevented the progression of %INT, which remained at similar levels as in Group Nx_pre_. No protection against interstitial expansion was obtained in rats treated with the AHHz association.

**Figure 8:**
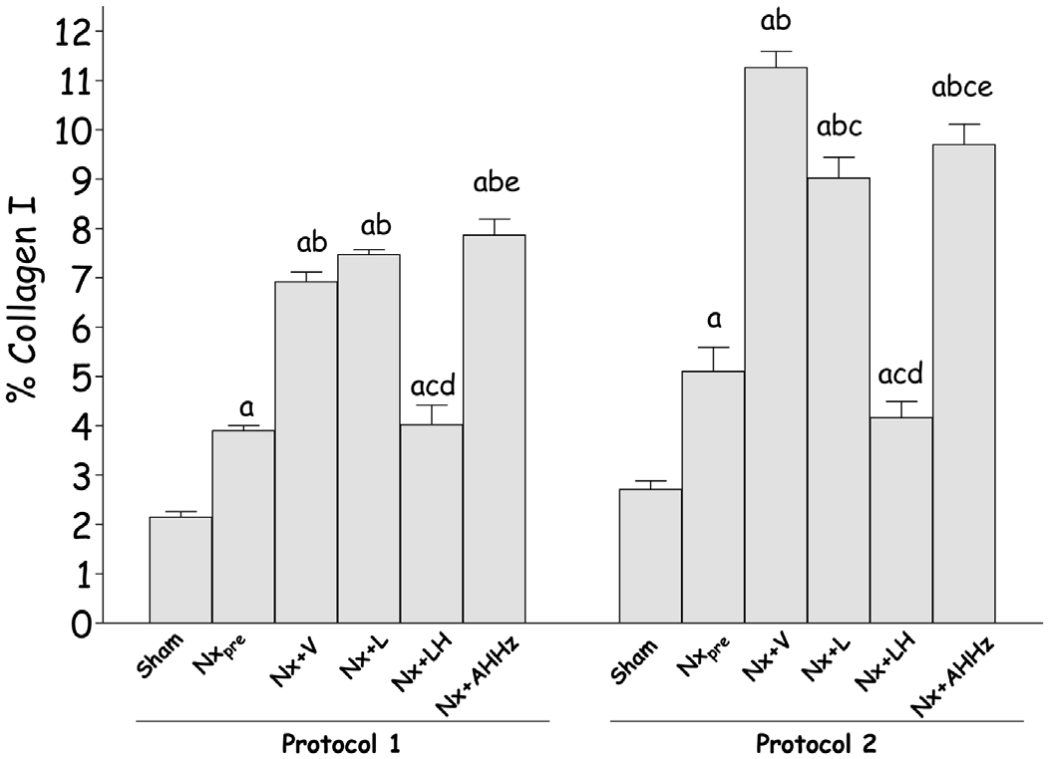
Quantitative analysis of percent renal area occupied by collagen I in Protocol 1 and Protocol 2. S, Sham-operated; Nx_pre_, pretreatment Nx (60 or 120 days after renal ablation); Nx+V, untreated Nx; Nx+L, losartan-treated Nx; Nx+LH, Nx treated with losartan+hydrochlorothiazide; Nx+AHHz, Nx treated with amlodipine, hydrochlorothiazide, and hydralazine. Results expressed as Mean ± SE. ^a^, p<0.05 vs. Sham; ^b^, p<0.05 vs. Nx_pre_; ^c^, p<0.05 vs. Nx+V; ^d^, p<0.05 vs. Nx+L and ^e^, p<0.05 vs. Nx+LH.

The presence of collagen I in the renal tissue, detected by immunohistochemistry, is shown in Fig. 5 (c, g, k, o, s, and w) for Protocol 1, and 6 (c, g, k, o, s, and w) for Protocol 2. The intensity of collagen 1 deposition (Fig. 8) paralleled the fraction of cortical area occupied by interstitial tissue, indicating that at least part of the interstitial expansion observed in Nx rats was due to fibrosis. No regression of collagen I deposition was seen with any of the treatments. However, renal interstitial fibrosis was arrested at pretreatment levels by the LH treatments in both Protocol 1 and 2.

**Figure 9:**
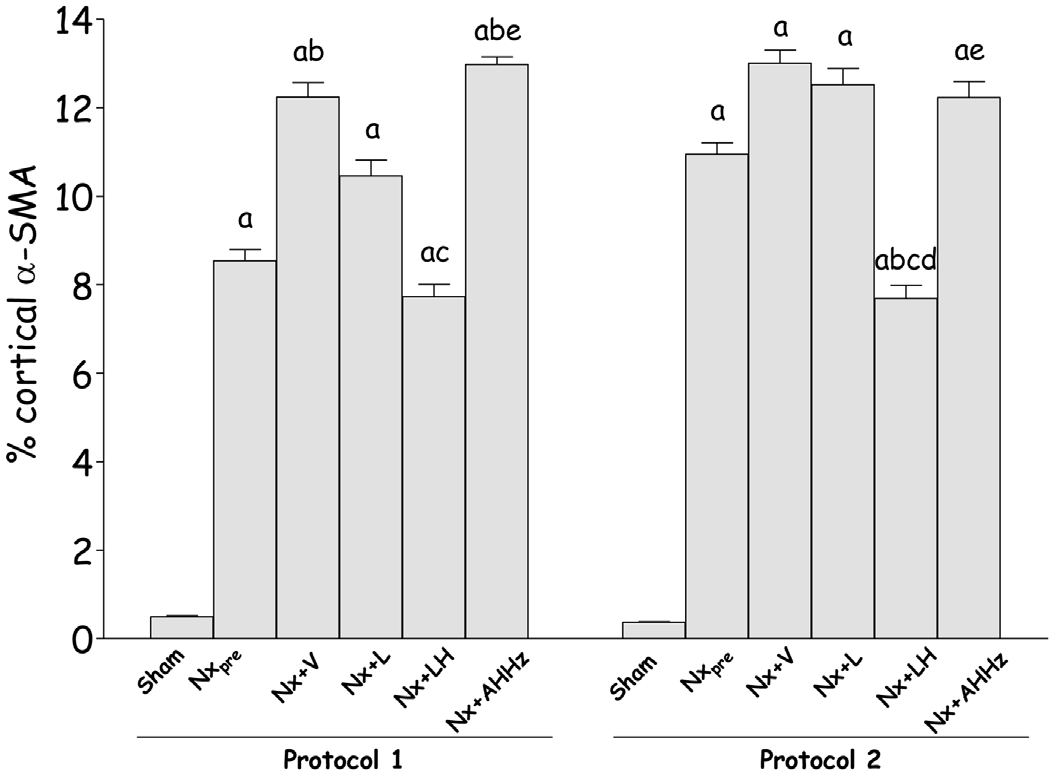
Percent cortical alpha-smooth muscle actin (α-SMA) in Protocol 1 and Protocol 2. S, Sham-operated; Nx_pre_, pretreatment Nx (60 or 120 days after renal ablation); Nx+V, untreated Nx; Nx+L, losartan-treated Nx; Nx+LH, Nx treated with losartan+hydrochlorothiazide; Nx+AHHz, Nx treated with amlodipine, hydrochlorothiazide, and hydralazine. Results expressed as Mean ± SE. ^a^, p<0.05 vs. Sham; ^b^, p<0.05 vs. Nx_pre_; ^c^, p<0.05 vs. Nx+V; ^d^, p<0.05 vs. Nx+L and ^e^, p<0.05 vs. Nx+LH.

Representative microphotographs of interstitial α-SMA obtained 150 days (Protocol 1) and 210 days (Protocol 2) after renal ablation are shown in Fig. 5 (d, h, l, p, t and x) and Fig. 6 (d, h, l, p, t and x), respectively. Figure 9 shows the fraction of the interstitial area occupied by α-SMA. In Protocol 1, pretreatment values (60 days after Nx) were significantly higher than in S, and continued to grow until 150 days post-Nx (p<0.05 vs. Nx_pre_). Rats treated with L only or with the AHHz association showed values that were similar to those seen in Group Nx+V (p>0.05), whereas combined LH treatment effectively prevented the increase of interstitial α-SMA with time (p>0.05 vs. Nx_pre_). Parallel results were observed in Protocol 2, with the only difference that the fractional α-SMA area at the end of the study was significantly smaller in Group Nx+LH than in Group Nx_pre_.

Table 1 shows data on macrophage infiltration for Protocol 1. Sixty days after Nx, the density of macrophages at the tubulointerstitial compartment was markedly elevated in Group Nx_pre_ compared to S (p<0.05). This parameter showed a progressive nature, reaching final values (150 days after ablation) that were significantly higher than those observed in Group Nx_pre_. L and LH treatments, but not the AHHz association, prevented the intensification of tubulointerstitial macrophage infiltration (p>0.05 vs. Nx_pre_). Data for Protocol 2 are shown in Table 2. Marked tubulointerstitial macrophage infiltration was observed in the pretreatment group (Nx_pre_) 120 days after renal ablation (p<0.05 vs. S), with progression to final values (210 days after Nx) that were significantly higher than in the Nx_pre_ group. All treatments prevented progression of macrophage infiltration, although this parameter was numerically lower in Group Nx+LH.

**Figure 10:**
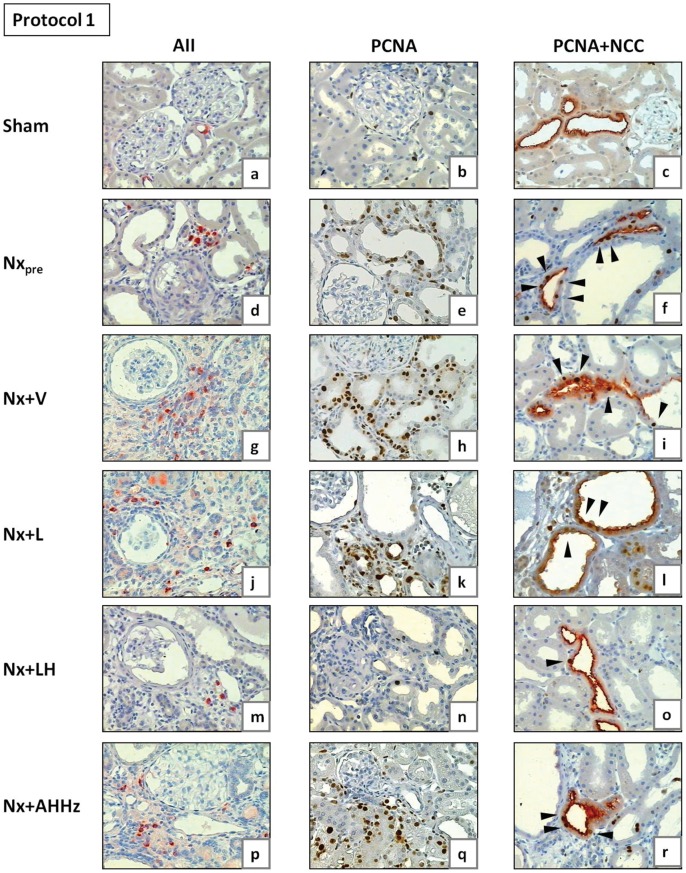
Representative microphotographs of renal tissue obtained for Nx_pre_ (60 days after renal ablation) and for all other groups (150 days after renal ablation) in Protocol 1. AII, tubulointerstitial cells staining positively for AII; PCNA, proliferating-cell nuclear antigen; NCC, sodium-chloride cotransporter, specific for distal convoluted tubule (DCT). Arrowheads in Figs. 10c, f, i, l, o and r (double staining for PCNA and NCC) indicate examples of PCNA-positive cells in DCT.

**Figure 11:**
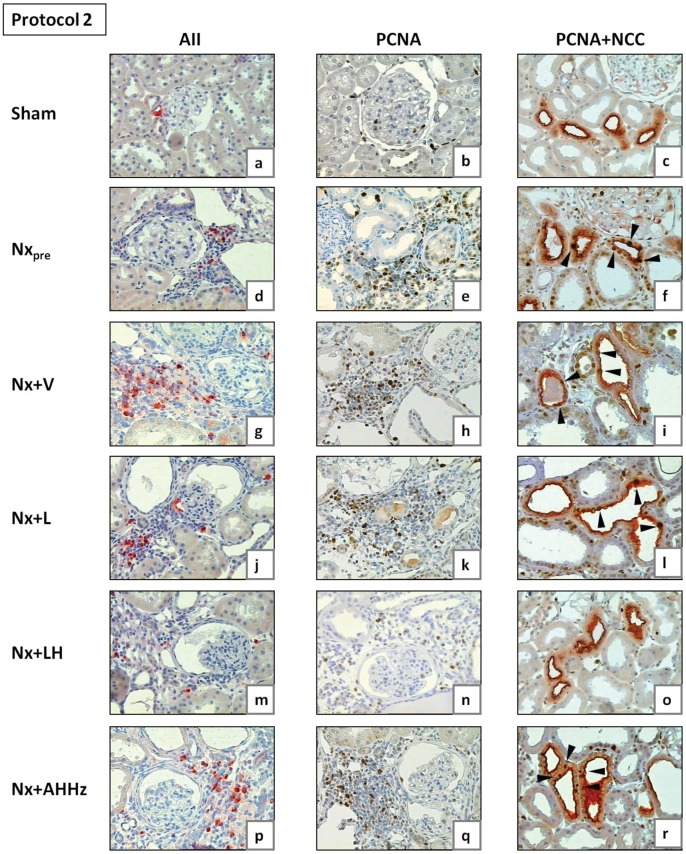
Representative microphotographs of renal tissue obtained for Nx_pre_ (120 days after renal ablation) and for all other groups (210 days after renal ablation) in Protocol 2. AII, tubulointerstitial cells staining positively for AII; PCNA, proliferating-cell nuclear antigen; NCC, sodium-chloride cotransporter, specific for distal convoluted tubule (DCT). Arrowheads in Figs. 11c, f, i, l, o and r (double staining for PCNA and NCC) indicate examples of PCNA-positive cells in DCT.

Representative microphotographs showing cells staining positively for AII are depicted in Fig. 10 (a, d, g, j, m and p) for Protocol 1, and in Fig. 11 (a, d, g, j, m and p) for Protocol 2. The density of AII-positive cells in Protocol 1 (Table 1) was already significantly higher than in S 60 days post-ablation (Group Nx_pre_), exhibiting further elevation 150 days after Nx (p<0.05 vs. Nx_pre_). Neither L monotherapy nor the AHHz association were able to prevent the increase in the number of AII-positive cells, while the LH combined therapy reduced this parameter to values that were similar to those found in Group Nx_pre_, and significantly lower than in each of the other Nx groups. Entirely parallel results were obtained in Protocol 2 (Table 2).

**Figure 12:**
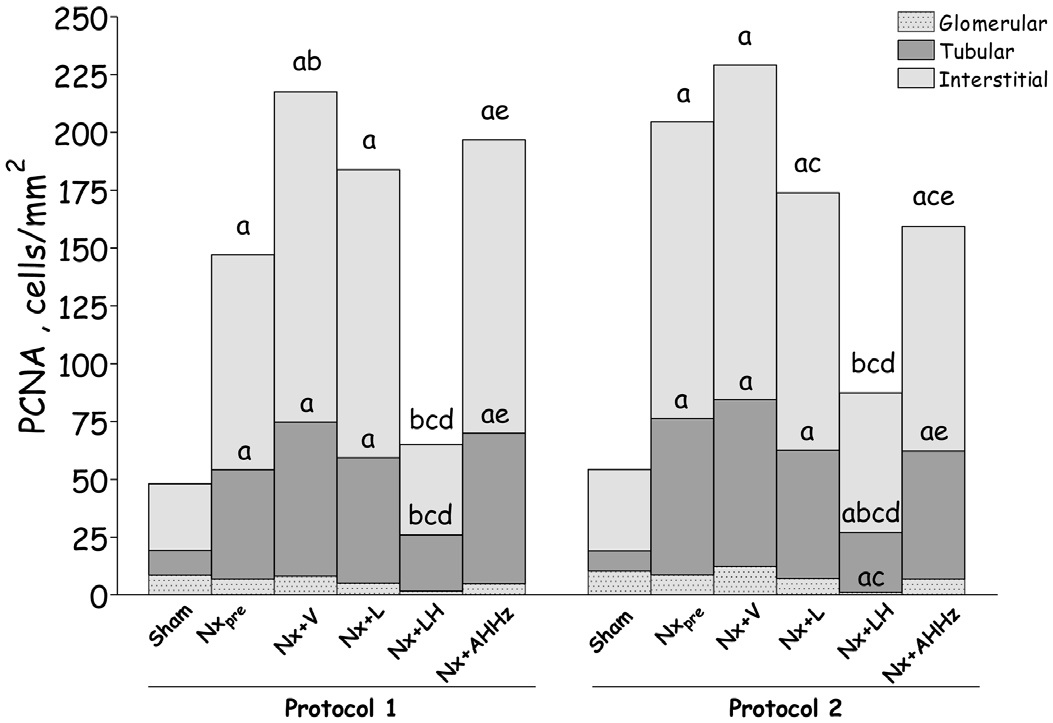
PCNA (Proliferating-cell nuclear antigen)-positive cells in glomerular (dotted areas), tubular (dark grey areas) and interstitial (light grey areas) compartments in Protocol 1 and Protocol 2. S, Sham-operated; Nx_pre_, pretreatment Nx (60 or 120 days after renal ablation); Nx+V, untreated Nx; Nx+L, losartan-treated Nx; Nx+LH, Nx treated with losartan+hydrochlorothiazide; Nx+AHHz, Nx treated with amlodipine, hydrochlorothiazide, and hydralazine. Results expressed as Mean ± SE. ^a^, p<0.05 vs. Sham; ^b^, p<0.05 vs. Nx_pre_; ^c^, p<0.05 vs. Nx+V; ^d^, p<0.05 vs. Nx+L and ^e^, p<0.05 vs. Nx+LH. Letters denoting significance are placed immediately above the corresponding bar area.

Representative microphotographs of renal proliferating cells are shown in Fig. 10 (b, e, h, k, n and q) for Protocol 1, and 11 (b, e, h, k, n and q) for Protocol 2. Figure 12 represents the quantitative data on cell proliferation, obtained by analysis of cells staining positively for PCNA. In Protocol 1, the intensity of cell proliferation in the tubular and interstitial compartments was significantly increased compared to S in the pretreatment group (Nx_pre_). Interstitial, but not tubular proliferation was significantly increased in untreated Nx rats compared to Nx_pre_. Among treatments, only the LH association was able to prevent, and even reverse, cell proliferation in both tubular and interstitial compartments, bringing these parameters to levels that were similar to those found in S. In Protocol 2, pretreatment values for tubular and interstitial proliferation were higher than in S. No progression of these parameters was seen in untreated Nx rats 210 days after renal ablation. At this time, interstitial proliferation was less intense in rats that received L monotherapy or the AHHz combined therapy than in Group Nx+V, but remained at levels similar to those observed prior to treatments. No difference was observed at this time among Groups Nx+V, Nx+L and Nx+AHHz regarding tubular cell proliferation. In rats treated with the LH association, values for PCNA-positive cells in all three compartments were significantly lower than in the Nx+V group, and even lower than in the pretreatment group, indicating that treatment promoted regression of cell proliferation in this group.

**Figure 13:**
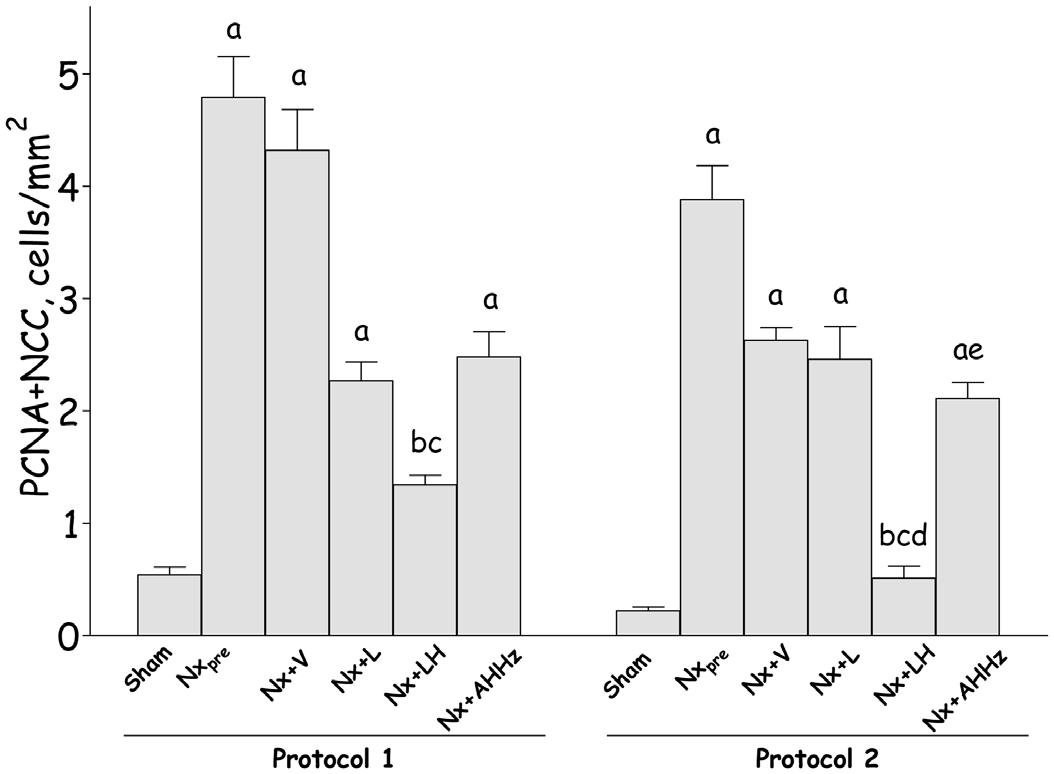
Frequency of proliferating cells in distal convoluted tubule, evaluated by double staining for PCNA (Proliferating-cell nuclear antigen) and NCC (distal convoluted tubule-specific sodium-chloride cotransporter) in Protocol 1 and Protocol 2. S, Sham-operated; Nx_pre_, pretreatment Nx (60 or 120 days after renal ablation); Nx+V, untreated Nx; Nx+L, losartan-treated Nx; Nx+LH, Nx treated with losartan+hydrochlorothiazide; Nx+AHHz, Nx treated with amlodipine, hydrochlorothiazide, and hydralazine. Results expressed as Mean ± SE. ^a^, p<0.05 vs. Sham; ^b^, p<0.05 vs. Nx_pre_; ^c^, p<0.05 vs. Nx+V; ^d^, p<0.05 vs. Nx+L and ^e^, p<0.05 vs. Nx+LH.

Representative microphotographs of proliferation in the DCT, obtained through simultaneous staining for PCNA and NCC, are shown in Fig. 10 (c, f, i, l, o and r) for Protocol 1, and Fig. 11 (c, f, i, l, o and r) for Protocol 2. The corresponding quantitative data are shown in Fig. 13. In Protocol 1, pretreatment DCT proliferation (60 days after renal ablation) was significantly increased compared with S. DCT proliferation remained elevated in untreated rats at the end of the study, with no progression compared to Nx_pre_. L monotherapy and the AHHz regimen promoted no significant change in DCT proliferation. By contrast, the LH association reduced DCT proliferation below pretreatment levels, indicating regression of this parameter. Parallel results were obtained in Protocol 2, with the exception that the differences between Group Nx+LH and either Nx+L and Nx+AHHz were now significant.

In Protocol 1 (Table 1), BG levels were unchanged in Group Nx_pre_ compared with S. At the end of the study (150 days after Nx) BG was slightly elevated in all groups, although only in Groups Nx+L and Nx+AHHz was this value significantly different from that in S. In Protocol 2 (Table 2), BG was again similar in Nx_pre_ and S. At the end of the study (210 days after renal ablation), BG was significantly increased in untreated Nx+V rats (p<0.05 vs. S). None of the treatments was able to normalize BG, which remained significantly increased compared to S in all groups of treated rats. Serum triglyceride concentrations (Tg) for Protocol 1 are given in Table 1. Tg was unaltered in the pretreatment group compared to S. Final Tg values were significantly increased in Group Nx+V (p<0.05 vs. S). Although L and AHHz treatments tended to normalize Tg, only the LH association was able to bring this parameter to values significantly lower than in the Nx+V group. In Protocol 2 (Table 2) Tg was significantly higher than S in the pretreatment group (Nx_pre_), and remained elevated at the end of the study. Only with the LH therapy did Tg fall to values significantly lower than in the Nx+V group, and similar to pretreatment values.

## Discussion

As described earlier [2]–[6], [15], reduction of renal mass in 5/6 resulted in systemic hypertension, heavy albuminuria, progressive glomerulosclerosis (GS), interstitial expansion/collagen deposition, marked tubulointerstitial proliferation, as well as interstitial infiltration by macrophages, myofibroblasts, and cells staining positively for AII. Mortality was very high in the untreated group, exceeding 40% at 150 days and 70% 210 days after nephrectomy, thus mimicking the picture observed in clinical practice. Serum aldosterone rose progressively in untreated Nx rats and, in view of its well-known profibrotic actions [21]–[23] likely made a substantial contribution to the progression of renal injury. Although the main factor stimulating the synthesis of aldosterone are circulating levels of AII, its production by the adrenals was probably influenced by potassium retention as well [24], helping to maintain the balance of this ion at the cost of worsening renal injury.

In most previous studies of the Nx model, treatments intended to prevent or ameliorate CKD were initiated immediately after nephrectomy or at most a few weeks after the procedure. At this initial phase, structural injury in the remaining renal tissue is incipient or only mild, and can be more easily prevented. We showed previously [25] that treatment with losartan and mycophenolate mofetil during the first 30 days after 5/6 nephrectomy strongly attenuated the progression of CKD, and that this protective effect persisted long after treatment was terminated. The same treatment had a much smaller impact when instituted between the 30th and 60th day after nephrectomy, indicating that early events determine the long-term outcome in this model. These findings may help explain the relative failure, in the clinical context, of treatments that appeared promising in experimental models.

In the present study, we compared the response of Nx rats to L monotherapy and the LH association, started at two different times after nephrectomy –60 and 120 days. Whereas the first time point simulated a condition of low nephron number with relatively mild renal inflammation, the second one –120 days after nephrectomy, never used before as a starting point for therapy – represented a situation in which drastic nephron reduction leads to high mortality and coexists with severe ongoing renal injury, with extensive glomerulosclerosis, interstitial inflammation and tubular atrophy, thus mimicking more closely the conditions prevailing in patients with advanced CKD, and their response to therapy.

Monotherapy with L provided only partial protection against mortality and the progression of renal injury/inflammation, especially when started 120 days after ablation. These results are consistent with previous observations [13], [26], and contrast sharply with studies in which L treatment, started concomitantly with renal ablation or shortly thereafter, exerted a much clearer renoprotective effect. Taken together, these findings strengthen the concept that, once set in motion, the mechanisms that operate in CKD can no longer be controlled by inhibition of the RAS only, requiring association with other drugs or procedures to be arrested.

In both protocols, the effectiveness of treatment with L was sharply increased when associated with H, promoting regression of U_alb_V and hypertension to levels below those observed before treatment, arresting the progression of renal injury and the decline of renal function, and dramatically reducing mortality. The factors that might explain this remarkable synergistic action are unclear. One obvious possibility is the striking BP reduction obtained with LH treatment [27], [28]. However, AHHz treatment promoted a similar decrease in BP without corresponding renoprotection (although it limited macrophage infiltration), indicating that the beneficial effects of L+H treatment did not derive from its antihypertensive effect. Intracapillary hypertension is another important pathogenic factor in the Nx model, promoting mechanical stress and associated inflammatory events [29]–[31]. In the present study, measurement of intraglomerular pressure was impossible due to the severe alteration of the renal surface, made extremely irregular by advanced disease. Since we showed previously that LH treatment started 30 days after Nx, unlike L monotherapy, completely reversed glomerular hypertension [15], it is plausible that a similar effect may have occurred in the present study. Another possible explanation for the renoprotection afforded by the LH combination is its dramatic action on plasma aldosterone, which in both protocols was reduced to levels lower than those observed before treatment and similar to those seen in S. Previous studies showed that blockade of the mineralocorticoid receptor attenuates chronic renal injury in the Nx model [32], [33], although inhibition of aldosterone synthesis has not been used in this context. Besides limiting fibrosis, normalization of aldosterone levels may have enhanced the natriuretic effect of H by inhibiting sodium reabsorption at principal cells and by a direct effect on NCC [34], thus exerting a protective effect by two distinct mechanisms. This strong inhibition of aldosterone production may have resulted from a synergistic interaction between L and H: on one hand, blockade of adrenal AT-1 receptors directly neutralized a major stimulus for the synthesis of aldosterone; on the other, H treatment, by facilitating kaliuresis, may have lessened a second important factor for aldosterone production. Finally, the antiproliferative effect of the LH association, bringing rates for glomerular, tubular and interstitial cell proliferation to levels lower than those observed at baseline, should be considered, since multiplication of local cells, including myofibroblasts and their precursors, is an important part of the inflammatory response triggered by renal mass removal [5], [35]. This striking effect may also reflect the observed regression of albuminuria, which may have removed the inflammatory stimulus of filtered protein to proximal tubular cells [36], [37]. Additionally, the remarkable reduction of DCT proliferation in the LH-treated group may have resulted from a local effect of H [38].

Insulin resistance and dyslipidemia may accompany CKD, aggravating renal injury [39]–[41]. Corroborating these clinical data, we observed an increase in plasma glucose and triglycerides in untreated Nx rats with advanced CKD (Protocol 2). Although thiazides may worsen these metabolic abnormalities [42], we did not observe such effects in association with LH treatment, which, on the contrary, reversed hypertriglyceridemia. Neither L monotherapy nor the AHHz regimen had any effect on circulating glucose or triglyceride levels, suggesting that the beneficial effect of the LH treatment on triglycerides resulted from the synergistic effect of the two drugs, perhaps reflecting amelioration of renal function.

Although thiazides have been used for decades in the treatment of hypertension, and although their value as antihypertensive agents in CKD has recently been affirmed [43], [44], a possible renoprotective effect of these compounds in CKD has not been systematically investigated. We showed previously that H monotherapy initiated 30 days after renal ablation attenuated systemic hypertension and renal injury without preventing their progression, an effect comparable to that obtained with L monotherapy [15]. Likewise, the association of thiazides with inhibitors of the RAS has not been evaluated as a possible resource against progression of advanced CKD, possibly as a corollary to the widespread notion that thiazides lack any effect at this phase. The present observations are once again at odds with this concept, which had already been challenged by the observations made in our laboratory [15], and by a few clinical studies [43], [45]. Thus, further studies focused on the possible effect of the L+H association on CKD progression are warranted.

In summary, the present results suggest that the L+H association arrests the progression of renal injury in the Nx model, even when initiated at advanced stages of the nephropathy, with ongoing GS and interstitial inflammation and fibrosis. Our results do not support the concept that thiazides lose their effect in advanced CKD, nor do they reproduce the metabolic effects attributed to chronic thiazide use. The renoprotective action of the LH association cannot be explained by its antihypertensive effect. Mechanisms possibly involved in this protective action are a fall in intraglomerular pressure, the sharp decrease observed in the filtration of albumin and in the production of aldosterone, as well as an antiproliferative effect. Additional studies are needed to investigate whether these renoprotective effects can also be obtained in human CKD even when treatment is initiated at very advanced stages.
